# Exploration of differentially-expressed exosomal mRNAs, lncRNAs and circRNAs from serum samples of gallbladder cancer and xantho-granulomatous cholecystitis patients

**DOI:** 10.1080/21655979.2021.1972780

**Published:** 2021-09-04

**Authors:** Jiajun Ren, Sheng Chen, Feng Ye, Xiaoyong Gong, Ye Lu, Qiang Cai, Yongjun Chen

**Affiliations:** Department of General Surgery, Shanghai Institute of Digestive Surgery, Ruijin Hospital, Shanghai Jiao Tong University, School of Medicine, Shanghai, China

**Keywords:** Gallbladder cancer, exosome, mRNA, lncRNA, circRNA

## Abstract

Gallbladder cancer (GBC) is the most common biliary tract malignancy worldwide. Although a growing number of studies have explored the mechanism of GBC, thus far, few molecules have been discovered that can be utilized as specific biomarkers for the early diagnosis and therapeutic treatment of GBC. Recent studies have shown that exosomes not only participate in the progression of tumors, but also carry specific information that can define multiple cancer types. The present study investigated the expression profiles of coding (or messenger) ribonucleic acids (mRNAs) and non-coding RNAs (ncRNAs, including long non-coding RNAs [lncRNAs] and circular RNAs [circRNAs]) in plasma-derived exosomes from GBC patients. Using high-throughput RNA sequencing and subsequent bioinformatic analysis, a number of differentially expressed (DE) mRNAs, lncRNAs, and circRNAs were identified in GBC exosomes, compared to their expressions in xantho-granulomatous cholecystitis (XGC) exosomes. Gene Ontology (GO) and Kyoto Encyclopedia of Gene and Genome (KEGG) analyses were then conducted to investigate the potential functions of these DE RNAs. Furthermore, the interaction networks and competing endogenous RNA networks of these DE RNAs and their target genes were investigated, revealing a complex regulatory network among mRNAs and ncRNAs. In summary, this study demonstrates the diagnostic value of plasma-derived exosomes in GBC and provides a new perspective on the mechanism of GBC.

## 1. Introduction

Gallbladder cancer (GBC) is the most aggressive malignancy of the biliary tract system; globally, it affects more than 219,420 people and causes 165,087 deaths every year [1]. Late diagnosis is a vital factor regarding poor outcomes for GBC; the five-year survival rate is less than 5%[2]. Most GBC patients are asymptomatic during the early stage. Furthermore, even following the development of late stage GBC, the symptoms exhibited can be similar to those of more benign diseases, such as cholecystitis, gallstone disease, and xantho-granulomatous cholecystitis (XGC). This may lead to a false negative diagnosis, leading to GBC patients missing the optimal chance for a complete surgical resection, which is currently the only known curative treatment for GBC [3]. Therefore, it is of great importance to identify effective biomarkers for the early diagnosis of GBC, and to improve its prognosis.

Exosomes are a category of small vesicles that range in size from to 30–150 nm; they exist in various body fluids, such as peripheral blood, urine, and bile. Exosomes are secreted by a broad spectrum of cells; they not only carry messages to recipient cells to mediate intercellular communication, but also the molecular characteristics of their parental cells [4]. Analysis of the genetic profiles of exosomes has been proven to be an effective and noninvasive method for improving the early diagnosis of malignancies, and for better predicting prognoses [5–7]. Though currently unknown, the genetic characteristics of exosomes derived from GBC patients may provide new insights into the mechanisms of the origin and progression of GBC, and could help to identify novel biomarkers for diagnosing it and predicting its prognosis.

In the past decades, non-coding ribonucleic acids (ncRNAs) have been shown to play critical roles in the regulation of tumor progression and metastasis. The long non-coding RNA (lncRNA) plasmacytoma variant translocation 1 (PVT1) can promote the progression of GBC through the miR-143/HK2 axis [8]. The circular RNA (circRNA) cicERBB2 can also regulate proliferation-associated 2 G4 (PA2G4)-dependent recombinant deoxyribonucleic acid (rDNA) transcription to participate in the tumor progression of GBC [9]. Together with messenger RNAs (mRNAs), these RNA cargos are abundant in exosomes and are involved in the complex regulation of cell-cell communications. Zheng et al. found that miR-182 was highly expressed in GBC, and that exosomal miR-182 could significantly promote the migration and invasion of GBC cells by inhibiting reversion-inducing-cysteine-rich proteins with Kazal motifs [10]. However, the potential roles of these exosomal mRNAs, lncRNAs, and circRNAs in the development of GBC remain unknown.

Therefore, this study aimed to identify effective biomarkers of GBC in serum exosomes, and to contribute to the current understanding of the mechanism of GBC development. To this end, differentially expressed (DE) exosomal mRNAs, lncRNAs, and circRNAs were examined in the plasma of patients with GBC. Furthermore, the relevant biological functions of these RNAs were investigated through Gene Ontology (GO) categories and Kyoto Encyclopedia of Genes and Genomes (KEGG) analysis, and a lncRNA-mRNA interaction network was constructed. These analyses and observations provide evidence of the diagnostic value of plasma-derived exosomes in GBC, and can contribute to the current understanding of the mechanism of GBC, which requires further exploration.

## 2. Materials and methods

### 2.1. Patients

All patients provided written informed consent to participate in the study. The study protocol was approved by the Ethics Committee of Ruijin Hospital of Shanghai Jiao Tong University. In total, five patients with GBC were recruited from Ruijin Hospital, Shanghai Jiao Tong University between January 2019 and December 2019. All patients were pathologically diagnosed with gallbladder adenocarcinoma, and were free of any other form of cancer. The patients included did not receive any type of anticancer treatment during the study. In addition, five patients with XCG with no history of cancer were enrolled as negative controls. For exosome isolation, peripheral blood specimens (4 ml) were collected intravenously in BD Vacutainer® Venous Blood Collection Tubes containing ethylenediaminetetraacetic acid (EDTA). Plasma was isolated and frozen immediately at −80°C until use. Blood collection and experiments were performed in accordance with the Declaration of Helsinki.

### 2.2. Isolation and identification of exosomes

Before extraction, the plasma samples were filtered using a 0.8 µm filter. Then, an exoEasy Maxi kit (Qiagen, Inc.) was used to isolate exosomes from these pre-treated samples. The exosome concentrations were measured according to the manufacturer’s instructions, using a Pierce™ BCA Protein Assay Kit (Thermo Fisher Scientific, USA).

Transmission electron microscopy (TEM) was conducted to determine the morphologies of the exosomes, as previously described [11]. After isolation, exosomes were fixed with 4% paraformaldehyde in equal volume and loaded onto carbon-formvar-coated grids, until exosomes were completely absorbed. The grids were then stained with 2% uranyl acetate for 10 min and with lead citrate for 5 min, at room temperature. Exosomes were visualized using a JEOL JEM-1400 transmission electron microscope (JEOL, USA).

Nanoparticle tracking analysis (NTA) was used to track the numbers and sizes of exosomes. The exosome concentration of was diluted with phosphate-buffered saline (PBS) to 10–50 ng·ml^−^[1] to maintain the number of objects per frame between 20 and 100. Exosome samples were injected into the sample chamber at room temperature, and were then detected using a Nanosight NS 300 system (NanoSight technology, Malvern, UK) with a 488 nm laser and a high‐sensitivity scientific complementary metal–oxide–semiconductor (sCMOS) camera. Measurements were performed in triplicate at camera setting 13, with an acquisition time of 30 s and a detection threshold setting of seven. The data were analyzed using NTA analytical software (version 2.3).

Flow cytometry was performed to detect the specific protein markers of the exosomes. After isolation, the exosome samples were re-suspended using filtered PBS. These samples were then incubated with the fluorescein isothiocyanate (FITC)-conjugated anti-tetraspanin antibodies CD63 and CD81 (BD Biosciences, USA) at 4°C for 50 min. The fluorescent signals attached to the exosomes were detected using a BD Accuri C6 flow cytometer (BD Biosciences, USA). Data were analyzed using CytExpert 2.0.

### 2.3. RNA sequencing

To prepare exosomal RNA for RNA sequencing, the exosome samples were lysed using TRIzol (Invitrogen) and the RNA content was extracted according to the manufacturer’s instructions. Next, total RNA samples (2 μg) were treated with an Epicenter Ribo-Zero rRNA Removal Kit (Illumina) and RNase R (Epicenter) to remove ribosomal RNA; they were fragmented to approximately 200 bp. Then, a NEBNext® UltraTM RNA Library Prep Kit for Illumina® (NEB) was used to construct the RNA-seq libraries. The quality of each library was controlled using an Agilent 2200 TapeStation (Agilent). Sequencing was conducted using an Illumina HiSeq 3000 by RiboBio Biotechnology Co. (Guangzhou, China). Detailed information is provided in the Supplementary Materials and Methods.

### 2.4. Bioinformatic analysis

To obtain effective clean reads, the raw RNA sequencing data were first filtered to remove low-quality reads and residual ribosomal RNA (rRNA). The clean reads were then mapped and removed using an RNA central database. The remaining reads were used for alignment and analysis. For each sample, the rRNA-removed reads were mapped to the reference genome using HISAT2. Next, HTseq was used to calculate the count values and RPM values of the RNAs in each sample, and the candidate RNAs were subjected to annotation and length analysis. Differential expression analysis of the RNA-seq was conducted using DESeq2 in R, based on a fold-change (FC) ≥ 2.0, and a p-value < 0.05 (t-test). Hierarchical clustering was used to distinguish gene-expression patterns among samples. Data are available from the Gene Expression Omnibus (GEO) GSE166915. GO and KEGG analyses were performed to determine the roles of the DE RNAs. To construct the interaction network of lncRNAs and mRNAs, the Pearson correlations were calculated between each lncRNA expression value and each mRNA expression value. In this manner, the potentially co-expressed mRNAs of each lncRNA were calculated, based on a Pearson correlation coefficient of > 0.8 or < −0.8 (for p < 0.05). In view of the low expression levels of circRNAs in serum exosomes, only those circRNAs that were expressed in at least two samples were used for subsequent analysis. Detailed information is provided in the Supplementary Materials and Methods.

### 2.5. Statistical analysis

Data were analyzed using a two-tailed Student’s t-test. Statistical significance was set at p < 0.05.

## 3. Results

### 3.1. Exploration of DE exosomal mRNAs, lncRNAs, and circRNAs from plasma samples of GBC and XGC patients

**Figure 1.**
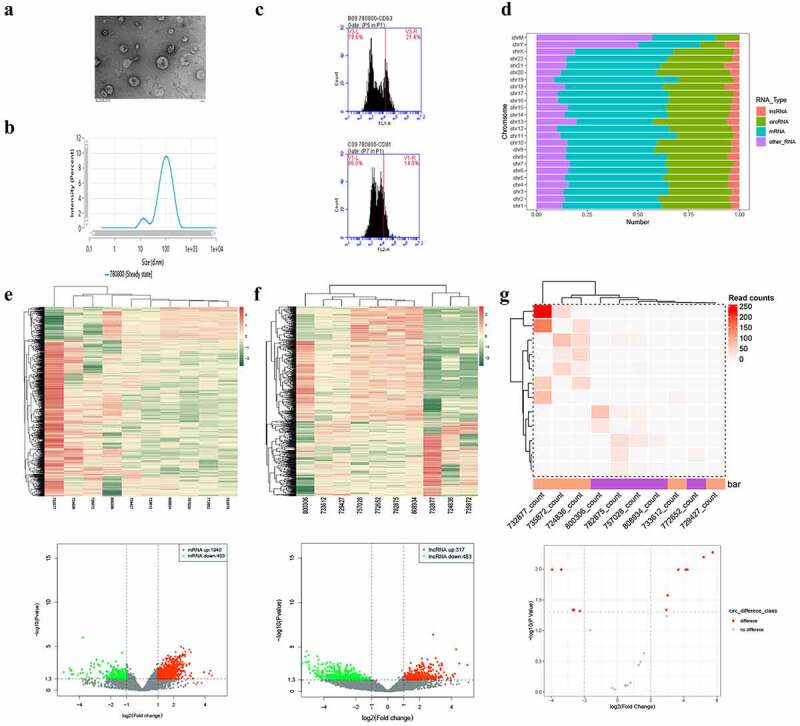
Analysis of differentially expressed exosomal RNAs between patients with gall bladder cancer and those with xantho-granulomatous cholecystitis. A-C. Identification of exosomes isolated from human plasma by transmission electron microscope, nanoparticle tracking analysis, and flow cytometry. D. Bar graph showing the gene distributions of human chromosomes. E-G. Heat maps and volcano plots of DE mRNAs, lncRNAs, and circRNAs

The isolation of plasma-derived exosomes was confirmed via TEM (Figure 1(a)), NTA (Figure 1(b)), and flow cytometry (Figure 1(c)), which were used to detect the biomarkers of exosomes. Approximately 21.4% and 14% of the collected samples were found to be positive for CD-63 and CD-81, respectively. Regarding the transcriptional characteristics of exosomes derived from the plasma samples of patients with GBC and XGC, transcriptome sequencing identified 45,006 mRNAs and 27,809 non-coding RNAs that were widely distributed across all chromosomes, including the sex chromosomes X and Y (Figure 1(d)). Among them, 1,940 mRNAs, 317 lncRNAs, and 7 circRNAs were upregulated in GBC plasma-derived exosomes, whereas 403 mRNAs, 483 lncRNAs, and 5 circRNAs were downregulated (Figure 1(e-g)). Hierarchical clustering analysis revealed that the expression profiles of exosomal DE mRNAs, lncRNAs, and circRNAs could distinguish GBC from XGC. Among these large numbers of significantly DE genes, a few genes, such as FOS, HMGA1, LCP1, PIM1, BCL2L2, TGFB1, MIR9-3HG, LRRC75A-AS1, LINC00969, ZNF503-AS1, NEAT1, LOC441204, circDNAJC6, and circPIK3C3, were found to be both widely expressed in every sample and significantly DE.

### 3.2. GO and KEGG enrichment analysis of DE mRNAs and target micro RNAs (miRNAs) of circRNAs

**Figure 2.**
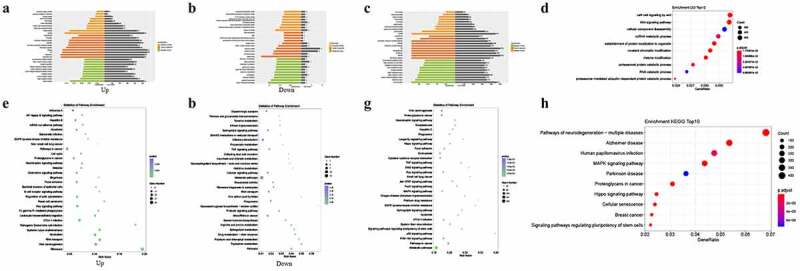
Enrichment analysis of differentially expressed (DE) mRNAs and circRNAs between gall bladder cancer and xantho-granulomatous cholecystitis. A, B. Gene Ontology (GO) analysis of DE mRNAs (upregulation and downregulation). C. GO analysis of DE lncRNAs. D. GO analysis of target miRNAs of DE circRNAs. E, F. KEGG analysis of DE mRNAs (upregulation and downregulation). G. KEGG analysis of DE lncRNAs. H. KEGG analysis of target miRNAs of DE circRNAs

CircRNAs are mainly generated from the exons or introns of parental genes shared with mRNAs. Recently, an increasing number of studies have reported that most circRNAs can function as competing endogenous RNAs (ceRNAs) and can regulate the expression of their parental genes. This suggests that the biological roles of circRNAs *in vivo* may be relevant to their parental genes. Based on this inference, the relationships between the expressions of circRNAs and their parental genes were analyzed. As shown in Figure S1, the expressions of screened circRNAs were independent from their parental genes, indicating that these circRNAs were eligible for subsequent study. GO categories and KEGG analyses of the functions of the target miRNAs of these DE mRNAs and circRNAs identified 2,168 and 54 GO terms annotated for upregulated and downregulated DE mRNAs, respectively (Figures S2-S7); the top 30 are presented in Figure 2(a,b). Furthermore, 4,094 GO terms were annotated for upregulated and downregulated DE lncRNAs (Figures S8-S10); the top 30 are presented in Figure 2(c). Regarding the target miRNAs of DE circRNAs, 3,408 GO terms were annotated (Supplementary Table S1); the top 10 GO terms are shown in Figure 2(d). Pathway analysis indicated that DE mRNAs were mainly involved in RNA transport, leukocyte trans-endothelial migration, the Ras signaling pathway, and drug metabolism (Figure 2(e,f)). DE lncRNAs were largely involved in metabolic pathways, including the mitogen-activated protein kinase (MAPK), PI3K-Akt, and Ras signaling pathways (Figure 2(g)). The target miRNAs of circRNAs were mostly enriched in the MAPK signaling pathway, and in proteoglycans in cancer (Figure 2(h)).

### 3.3. Identification and enrichment analysis of lncRNA-targeted mRNAs

**Figure 3.**
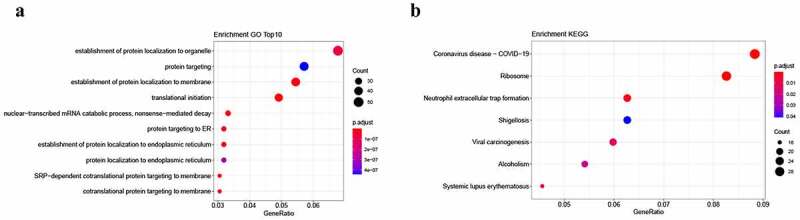
Enrichment analysis of differentially expressed lncRNA-targeted genes. a. Gene Ontology (GO) analysis. b. Kyoto Encyclopedia of Gene and Genome (KEGG) analysis

LncRNA-targeted mRNAs were analyzed to detect the possible biological functions of lncRNAs that were DE between GBC and XGC. Cis- (10 kb) and trans-regulation (e < 1E^−^[5]) analyses were conducted to identify the potential targets of DE lncRNAs, indicating that one lncRNA may be correlated with numerous mRNAs. Furthermore, these results revealed that one mRNA can be associated with numerous lncRNAs. As shown in Figure 3(a), GO analysis revealed that a total of 67 GO terms were annotated for mRNAs that were co-expressed with DE lncRNAs, including translational initiation and the establishment of protein localization to the membrane (Figures S11-S13). KEGG pathway analysis showed that the most relevant pathways for DE lncRNA-targeted mRNAs were ribosome and viral carcinogenesis pathways (Figure 3(b).

### 3.4. LncRNA-mRNA interaction network

**Figure 4.**
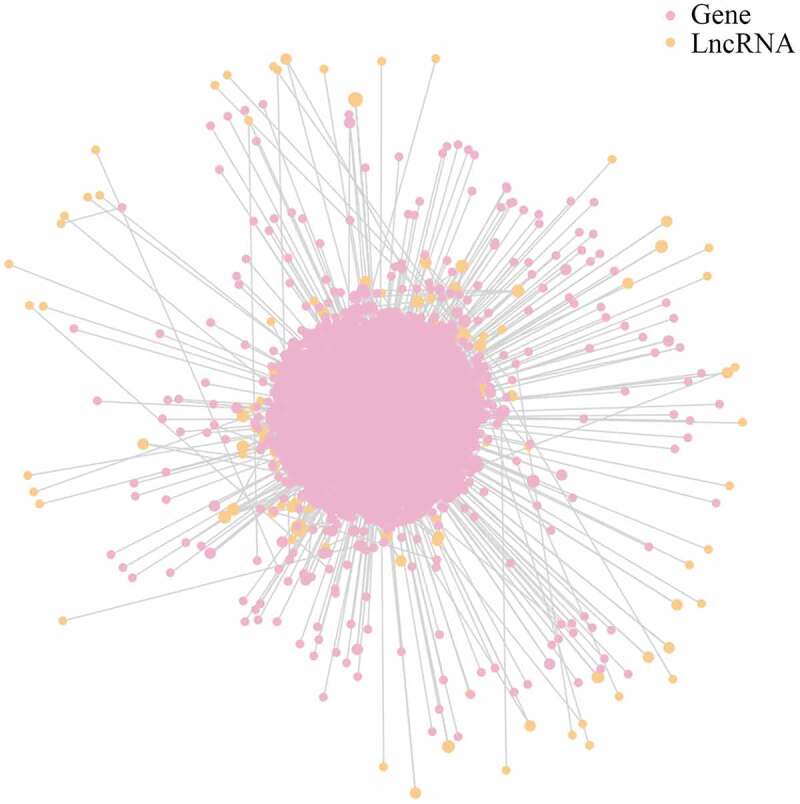
LncRNA-mRNA interaction network for plasma-derived exosomes of patients with gall bladder cancer

The present study identified 2,684 putative mRNAs for DE lncRNAs. An lncRNA-mRNA network was constructed to aid analysis of the interaction between lncRNAs and their target mRNAs. As shown in Figure 4 and Table S2, ENST00000435597.1 was found to be the most prominent and crucial RNA in this network, as it had the most targets.

### 3.5. CeRNA network between circRNAs and miRNAs

**Figure 5.**
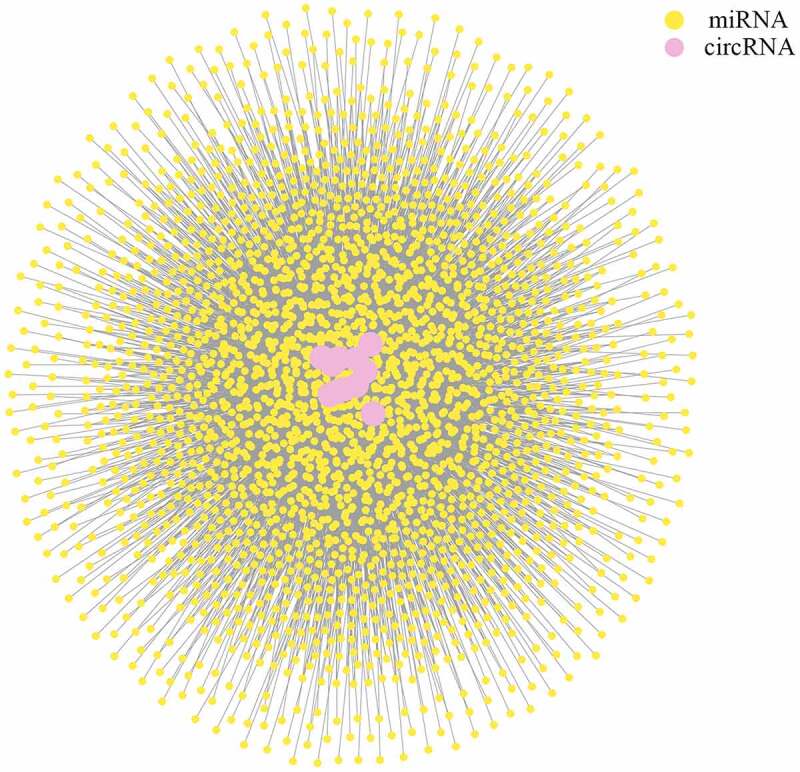
CeRNA network of circRNAs and miRNAs for plasma-derived exosomes of patients with gall bladder cancer

Sponging miRNAs and shedding their functions is one of the most important roles of circRNAs. To determine the potential roles of DE circRNAs, three software packages (miRanda, RNAhybrid, and TargetScan) were used to predict the miRNA targets of circRNAs working as ceRNAs. The resulting interaction network (Figure 5) showed the complicated regulatory network between circRNAs and miRNAs (Table S3).

### 3.6. Construction of protein-protein interaction network

**Figure 6.**
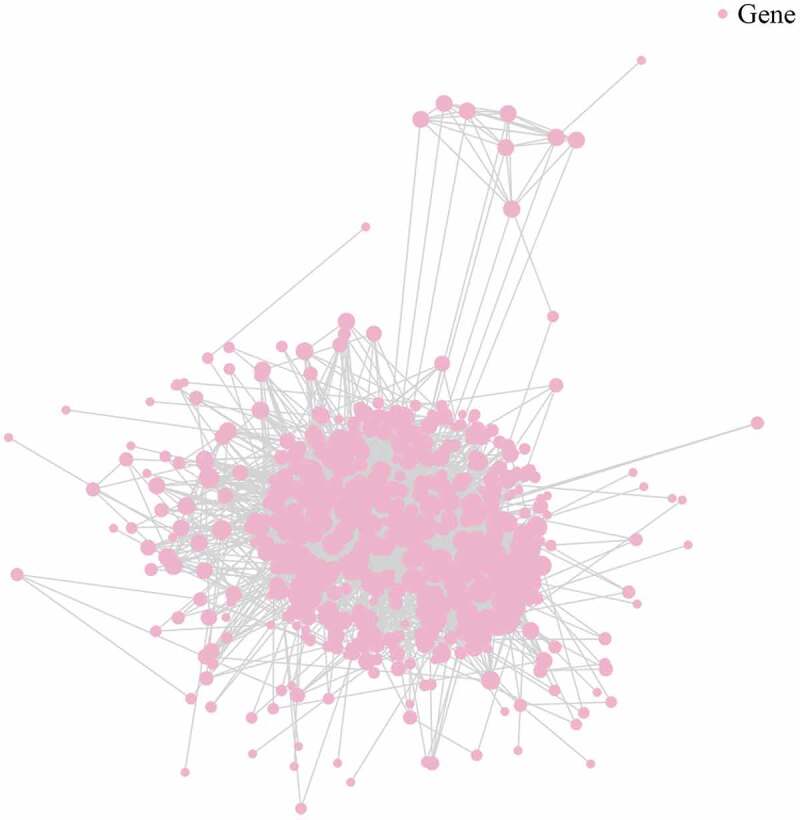
Illustration of protein-protein network for plasma-derived exosomes of patients with gall bladder cancer

Biological processes comprise numerous signaling pathways that are primarily executed by proteins. The precise interactions between these proteins generate a vast network that is closely linked to the functions it may present. To better understand the effect of these cancer-derived exosomes, a protein-protein interaction network was constructed by using the data of DE mRNAs and consulting several protein interaction databases [12] (Figure 6, Table S4).

## 4. Discussion

During the past decades, scientists and surgeons have worked together to improve the prognosis of GBC. One of the most crucial aspects regarding a good outcome is the early detection of GBC. It is difficult to achieve early detection using traditional methods, which are largely dependent on the experience of radiologists, ultrasonic examinations, computed tomography scans, or magnetic resonance imaging. A tissue biopsy is the only way to confirm a diagnosis of GBC. However, as an invasive operation, it inevitably has some complications, such as hemorrhages, infections, and the possibility of inducing metastasis [13,14]. Therefore, more and more attention is being paid to a newly emerging noninvasive method called ‘liquid biopsy’, the samples of which could contain the gene profile representing a specific cancer.

Cancer-derived exosomes form part of a liquid biopsy, and an accumulating growing body of evidence is revealing their non-negligible roles in the processes of tumor growth, invasion, and metastasis [15–19]. These exosomes carry specific materials that are released by cancer cells and cancer-associated stroma cells; they can indicate the characteristics of individual cancers, including their stage, metastasis, drug resistance, and recurrence. A panel of exosomal proteins has been reported to be able to distinguish tumor tissue from non-tumor tissue, with a sensitivity and specificity that are both over 90%[4]. The gene-expression of the RNA human telomerase reverse transcriptase (hTERT), as detected from plasma-derived exosomes, was found to induce the proliferation of pancreatic cancer by increasing telomerase activity [20]. Additionally, the dysregulation of circ-PDE8A in exosomes has been found to contribute to the invasive growth of pancreatic cancer through the MACC/MET/ERK/Akt signaling pathway [21]. As it is convenient to collect exosomes through various biofluids, such as plasma, urine, bile, and milk, the discovery of specific biomarkers exist in exosomes could lead to new methods for cancer diagnosis, treatment, and prognosis prediction. However, few studies [22] have investigated the transcriptional characteristics of GBC-derived exosomes. Zheng et al. reported that exosomal miR-182 was highly expressed in GBC and promoted the migration and invasion of GBC by inhibiting reversion-inducing-cysteine-rich protein with Kazal motifs (RECK), which could promote apoptosis and inhibit cell proliferation, migration, and invasion [10]. These studies have demonstrated the effective role of exosomal RNAs; however, the expression profiles of mRNAs, lncRNAs, and circRNAs in plasma exosomes from patients with GBC are still unknown.

Zhou et al. analyzed six original sequencing data of gastric cancer on the Illumina HumanHT-12 platform from the Array Expression and Gene Expression Omnibus, identifying 109 upregulated genes and 271 downregulated genes; they also constructed a new prognostic signature for gastric cancer [23]. Unlike said report, the present study utilized liquid biopsy to perform new sequencing, which is more innovative. In the present study, comparison with XGC-derived exosomes permitted the investigation of the expression characteristics of coding RNAs and ncRNAs in GBC-derived exosomes. The expression profiles revealed 2,303 DE mRNAs, 800 DE lncRNAs, and 12 DE circRNAs in GBC-derived exosomes, including 1,940 upregulated mRNAs, 403 downregulated mRNAs, 317 upregulated lncRNAs, 483 downregulated lncRNAs, 7 upregulated circRNAs, and 5 downregulated circRNAs. Functional annotation and enrichment analysis of DE mRNAs, DE lncRNA-targeted genes, and DE circRNA-parental genes was also conducted. GO analysis indicated that these DE genes were mainly correlated with the regulation of cellular metabolic processes, eosinophil migration, and eosinophil chemotaxis, as well as with the modification of histones and chromatin. Pathway analysis showed that these DE genes were significantly enriched in several cancer-related pathways, such as the PI3K-Akt signaling pathway, metabolic pathways, the p53 signaling pathway, the TGF-beta signaling pathway, and pathways regulating the pluripotency of stem cells. The PI3K-Akt pathway is one of the most classic pathways involved in the progression of several cancers; it regulates their growth, invasion, metastasis, and therapy resistance [4,24,25]. These differentially regulated biological processes in GBC-derived exosomes indicate the important roles of exosomes in the prediction of GBC. However, further research is needed to determine the roles and detailed mechanisms of these specific genes in regulating the progression of GBC, as well as to verify the importance of these gene hubs using large numbers of clinical samples. Nepal et al. analyzed 92 GBC whole-exome sequencing tumor/normal pairs; they annotated 3,845 single nucleotide variants and 432 small insertion/deletions, including TP53, ELF3, ERBB2, CDC27, TGFBR2, PIK3CA, KIR2DL4, KIR2DL3, and ARID2 [26]. Pandey et al. performed a larger sample size analysis of 167 GBC patients through whole-exome sequencing; they identified 25 significantly mutated GBC genes, including CTNNB1, ELF3, TP53, ERBB2, ARID2, ERBB3, STK11, CDKN2A, SMAD4, ARID1A, KRAS, EHF, PIK3CA, BRAF, ACVR2A, PSIP1, NFE2L2, CHRM3, ZNF107, SMARCA4, APC, NF1, KAT8, MAP2K4, and HIST1H2AG [27]. Unlike in these two studies, no differential expressions of these genes were observed in serum exosomes in the present study. Naturally, loci mutations do not necessarily affect gene expressions. However, there might also be several other reasons for this, including differences in sample size, sources of tissues, and serum exosomes.

## 5. Conclusion

The present study screened and analyzed the transcriptional expression profiles of GBC-derived serum exosomes using an RNA sequencing approach. This revealed a set of DE mRNAs, lncRNAs, and circRNAs, as well as their associated biological functions. This study provides new insights into the functions of coding RNAs and ncRNAs in exosomes derived from GBC, and reveals the clinical value of exosomes as part of a liquid biopsy approach to the diagnosis of GBC.

## Supplementary Material

Supplemental Material

## Data Availability

Data and analyses can be obtained from the corresponding author on a reasonable request. Sequence data are available from the GEO (GSE166915).

## Highlights

(1) A total of 1,940 mRNAs, 317 lncRNAs, and 7 circRNAs were upregulated in GBC plasma-derived exosomes, whereas 403 mRNAs, 483 lncRNAs, and 5 circRNAs were downregulated.

(2) 2,168 and 54 GO terms annotated for upregulated and downregulated DE mRNAs.

(3) 4,094 GO terms were annotated for upregulated and downregulated DE lncRNAs.

(4) 3,408 GO terms were annotated for the target miRNAs of DE circRNAs.

(5) DE mRNAs, lncRNAs, and circRNAs were involved in various tumor-associated signaling pathways.
