# Supplementary material for: Exploration of differentially-expressed exosomal mRNAs, lncRNAs and circRNAs from serum samples of gallbladder cancer and xantho-granulomatous cholecystitis patients
Source: Bioengineered. 2021 Sep 4;12(1):6134–43. doi: 10.1080/21655979.2021.1972780 (PMC8806659; doi:10.1080/21655979.2021.1972780)
Supplement: Supplemental Material [file KBIE_A_1972780_SM6899.zip › supplementary/Supplementary figure legends.docx]

**Supplementary figure legends**

Figure S1. Box plots showing the distributions of circRNAs and expression profiles of their parental genes.

Figure S2. GO analysis of molecular functions of upregulated mRNAs.

Figure S3. GO analysis of cellular components of upregulated mRNAs.

Figure S4. GO analysis of biological processes of upregulated mRNAs.

Figure S5. GO analysis of molecular functions of downregulated mRNAs.

Figure S6. GO analysis of cellular components of downregulated mRNAs.

Figure S7. GO analysis of biological processes of downregulated mRNAs.

Figure S8. GO analysis of molecular functions of DE lncRNAs.

Figure S9. GO analysis of cellular components of DE lncRNAs.

Figure S10. GO analysis of biological processes of DE lncRNAs.

Figure S11. GO analysis of molecular functions of DE lncRNA-targeted mRNAs.

Figure S12. GO analysis of cellular components of DE lncRNA-targeted mRNAs.

Figure S13. GO analysis of biological processes of DE lncRNA-targeted mRNAs.
