# Supplementary material for: Exploration of differentially-expressed exosomal mRNAs, lncRNAs and circRNAs from serum samples of gallbladder cancer and xantho-granulomatous cholecystitis patients
Source: Bioengineered. 2021 Sep 4;12(1):6134–43. doi: 10.1080/21655979.2021.1972780 (PMC8806659; doi:10.1080/21655979.2021.1972780)
Supplement: Supplemental Material [file KBIE_A_1972780_SM6899.zip › supplementary/Supplementary Materials and Methods.docx]

***Library construction and sequencing***

Total RNA was extracted from cell tissues using the Trizol (Invitrogen) according to the manufacturer’s protocol, and ribosomal RNA was removed using the Ribo-Zero™ kit (Epicentre, Madison, WI, USA). Fragmented RNA (the average length was approximately 200 bp) were subjected to first strand and second strand cDNA synthesis following by adaptor ligation and enrichment with a low-cycle according to instructions of NEBNext^®^ Ultra™ RNA Library Prep Kit for Illumina (NEB, USA). The purified library products were evaluated using the Agilent 2200 TapeStation and Qubit^®^2.0 (Life Technologies, USA). The libraries were paired-end sequenced (PE150, Sequencing reads were 150 bp) at Guangzhou RiboBio Co., Ltd. (Guangzhou, China) using IlluminaHiSeq 3000 platform with 2 × 150 bp mode.

***Pre-processing of sequencing reads/Quality Control***

1) Raw fastq sequences were treated with Trimmomatic tools (v0.36) using the following options TRAILING20, MINLEN235 and CROP235, to remove trailing sequences below a phred quality score of 20 and to achieve uniform sequence lengths for downstream clustering processes.

2) Sequencing read quality was inspected using the FastQC software. Adapter removal and read trimming were performed using Trimmomatic. Sequencing reads were trimmed from the end (base quality less than Q20) and filtered by length (less than 25).

***Quantification of gene expression level***

Paired-end reads were aligned to the mouse reference genome mm10 with HISAT2. HTSeq v0.6.0 was used to count the reads numbers mapped to each gene. The whole samples expression levels were presented as RPKM (expected number of Reads PerKilobase of transcript sequence per Million base pairs sequenced), which is the recommended and most common method to estimate the level of gene expression.

***Identification of new lncRNA***

The raw data were first filtered to remove low-quality reads, then the clean data that passed repeated testing was assembled using the StringTie based on the reads mapped to the reference genome. The assembled transcripts were annotated using gffcompare program. The unknown transcripts were used to screen for putative lncRNAs. Putative protein-coding RNAs were filtered out using a minimum length and exon number threshold. Transcripts with lengths above 200 nt with predicted ORF shorter than 300 nt were selected as lncRNA candidates. They were subjected to further screening using CPC/CNCI/Pfamto distinguish the protein-coding genes from the noncoding genes.

***Identification and quantification of circRNAs***

Two algorithms, CIRI2 and CIRCexplorer2 were used to detect circRNAs. Reads were mapped to human reference genome GRCh37/hg19 (http://genome.ucsc.edu/) by BWA-MEM or Tophat, respectively. CIRI2 detects the paired chiastic clipping (PCC) signals from the mapping information of reads by local alignment with BWA-MEM and combines with systematic filtering steps to remove potential false positives. CIRCexplorer2 uses TopHat and TopHat-Fusion alignment output to detect circRNAs. If a circRNA can be detected by both methods, it will be considered as an identified circRNA. Back-spliced junction reads identified in CIRI2 were combined and scaled to RPM (Reads Per Million mapped reads, BWA-MEM mapping) to quantify every circRNAs.

***Differential expression analysis***

The statistically significant DE genes were obtained by an adjusted P-value threshold of 0.05 and log2 (fold change)>1 using the DEGseq software. Finally, a hierarchical clustering analysis was performed using the R language package gplots according to the RPKM values of differential genes in different groups. And colors represent different clustering information, such as the similar expression pattern in the same group, including similar functions or participating in the same biological process.

***GO terms and KEGG pathway enrichment analysis***

All differentially expressed mRNAs were selected for GO and KEGG pathway analyses. GO was performed with KOBAS3.0 software. GO provides label classification of gene function and gene product attributes (http://www.geneontology.org). GO analysis covers three domains cellular component (CC), molecular function (MF) and biological process (BP). The differentially expressed mRNAs and the enrichment of different pathways were mapped using the KEGG pathways with KOBAS3.0 software (http://www.genome.jpkegg).

***Co-expression network of differentially expressed lncRNAs/mRNAs***

To investigate the potential functions of differentially expressed lncRNAs and the interactions between mRNAs and lncRNAs, we constructed alncRNA/mRNA transcripts co-expression network. The co-expression network was constructed by calculating the Pearson correlation coefficient and P value between multiple genes.
