# Supplementary figures and images for: Exploration of differentially-expressed exosomal mRNAs, lncRNAs and circRNAs from serum samples of gallbladder cancer and xantho-granulomatous cholecystitis patients
Source: Bioengineered. 2021 Sep 4;12(1):6134–43. doi: 10.1080/21655979.2021.1972780 (PMC8806659; doi:10.1080/21655979.2021.1972780)

A

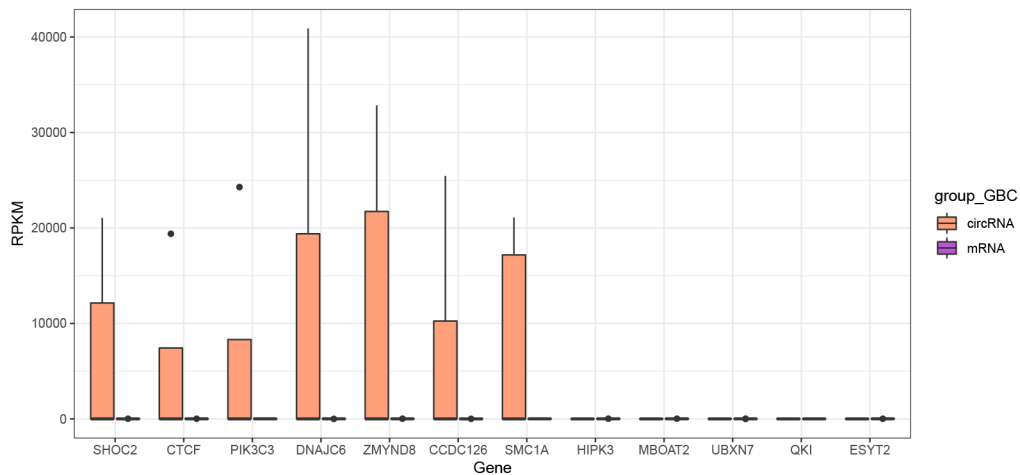

B

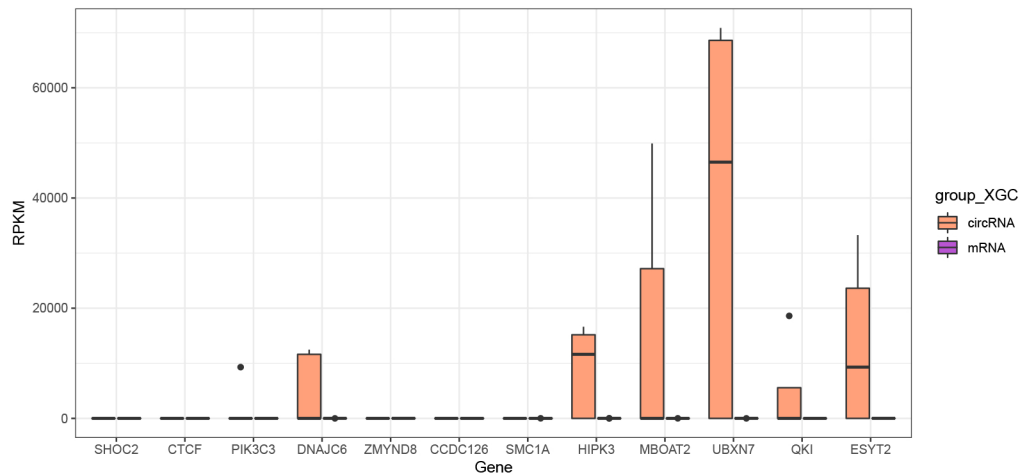

Supplement: Supplemental Material [file KBIE_A_1972780_SM6899.zip › supplementary/Supplementary Figure S1.pdf]

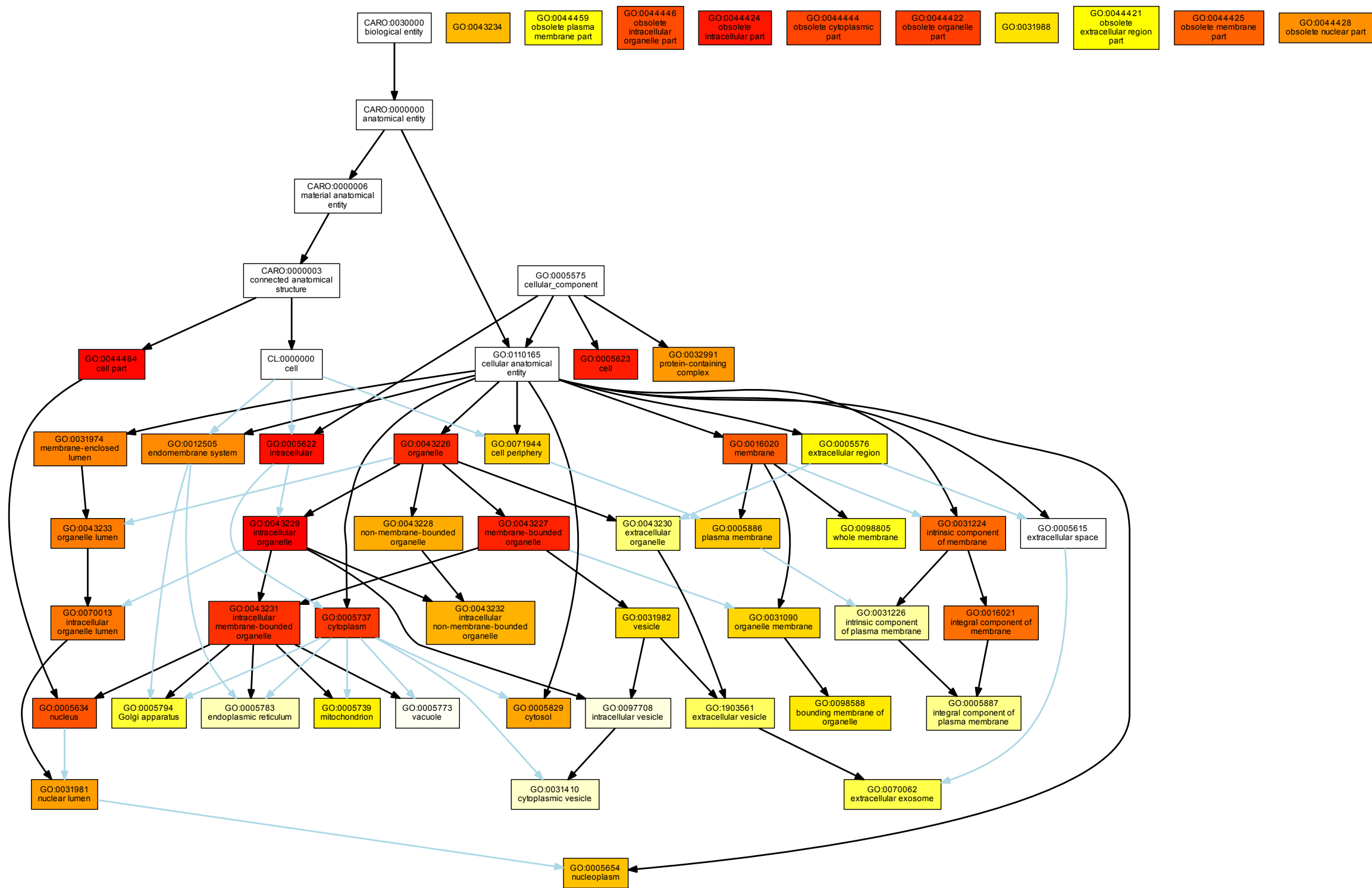

Supplement: Supplemental Material [file KBIE_A_1972780_SM6899.zip › supplementary/Supplementary Figure S12.pdf]

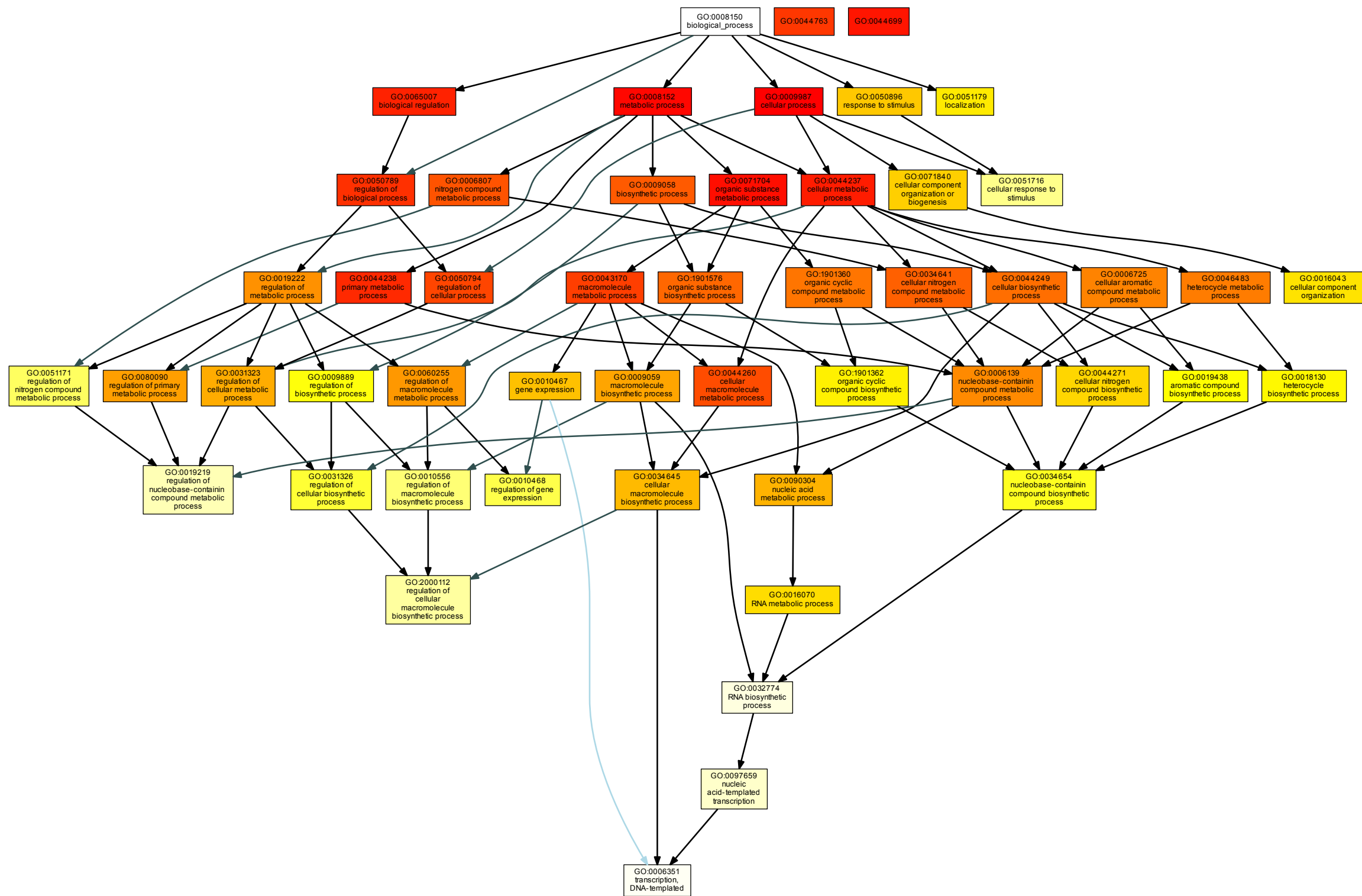

Supplement: Supplemental Material [file KBIE_A_1972780_SM6899.zip › supplementary/Supplementary Figure S13.pdf]

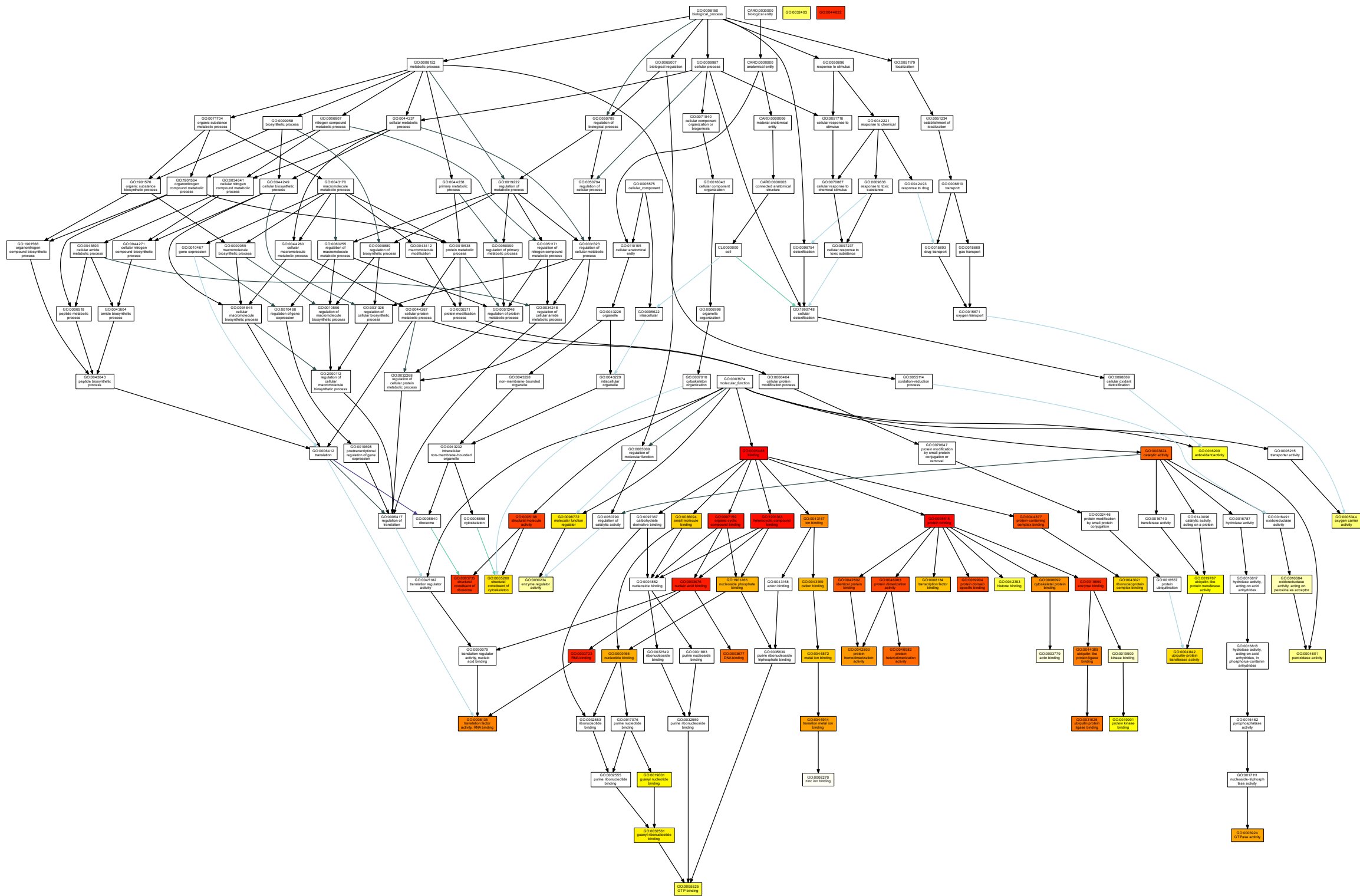

Supplement: Supplemental Material [file KBIE_A_1972780_SM6899.zip › supplementary/Supplementary Figure S2.pdf]

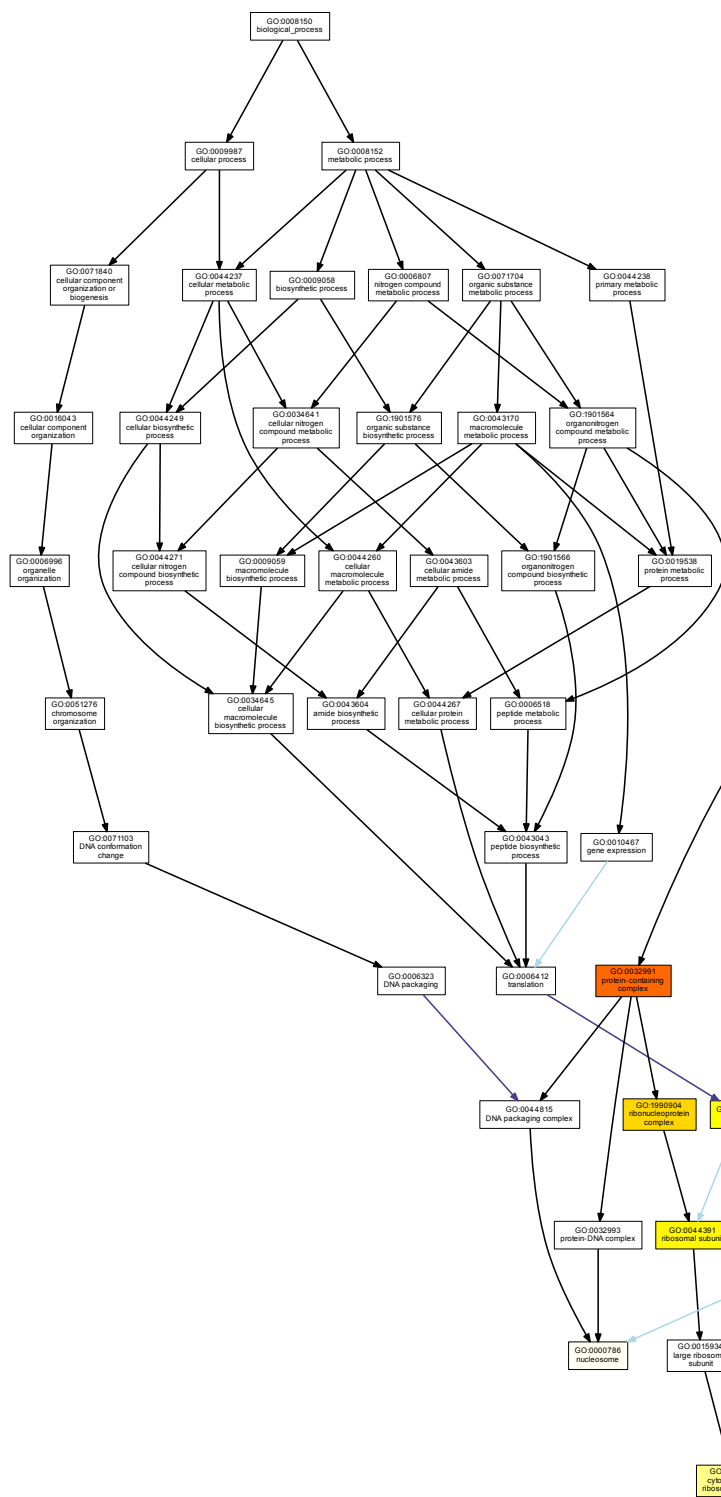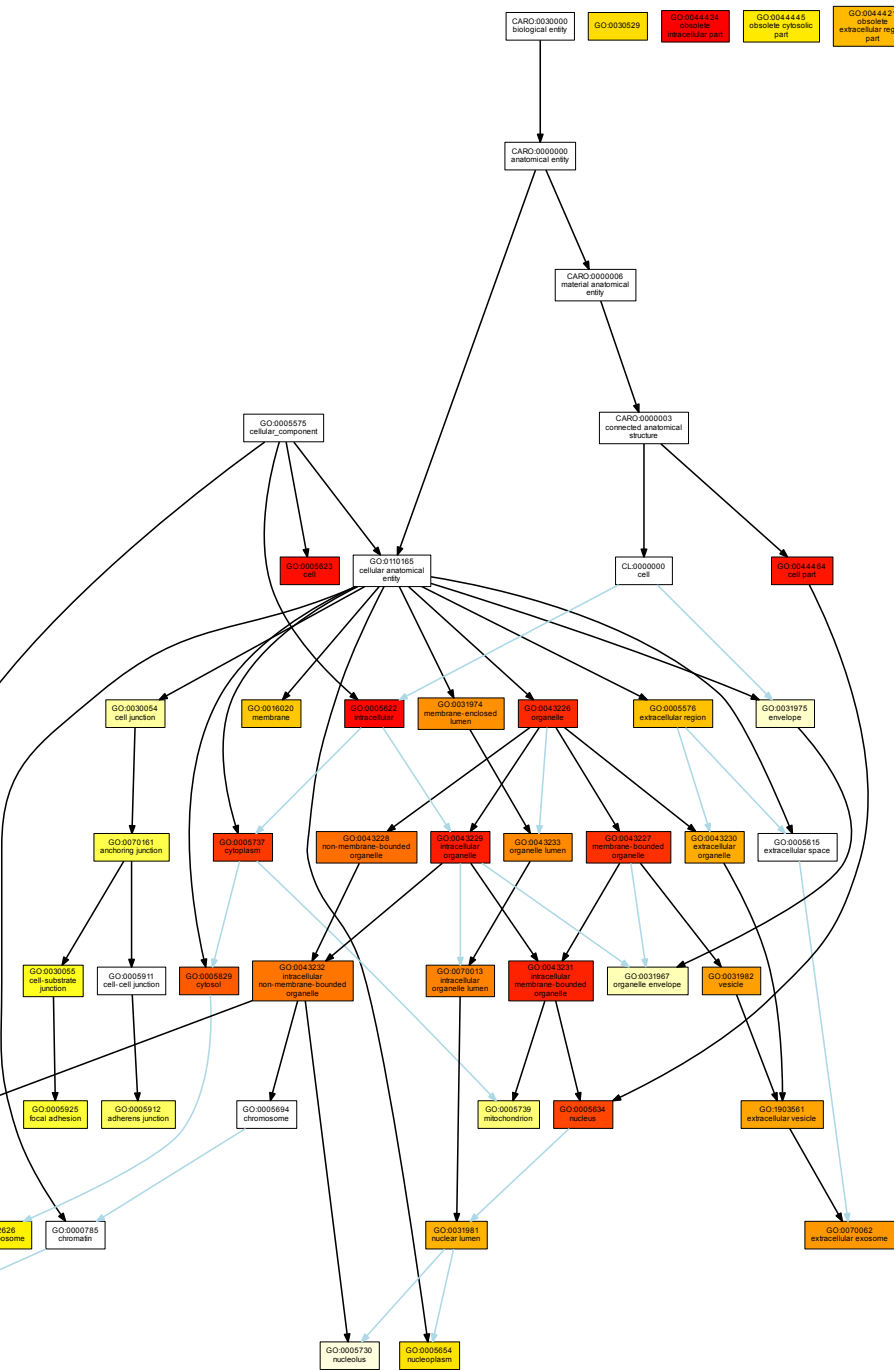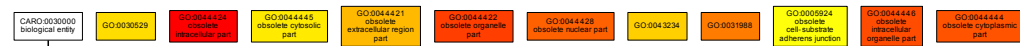

Supplement: Supplemental Material [file KBIE_A_1972780_SM6899.zip › supplementary/Supplementary Figure S3.pdf]

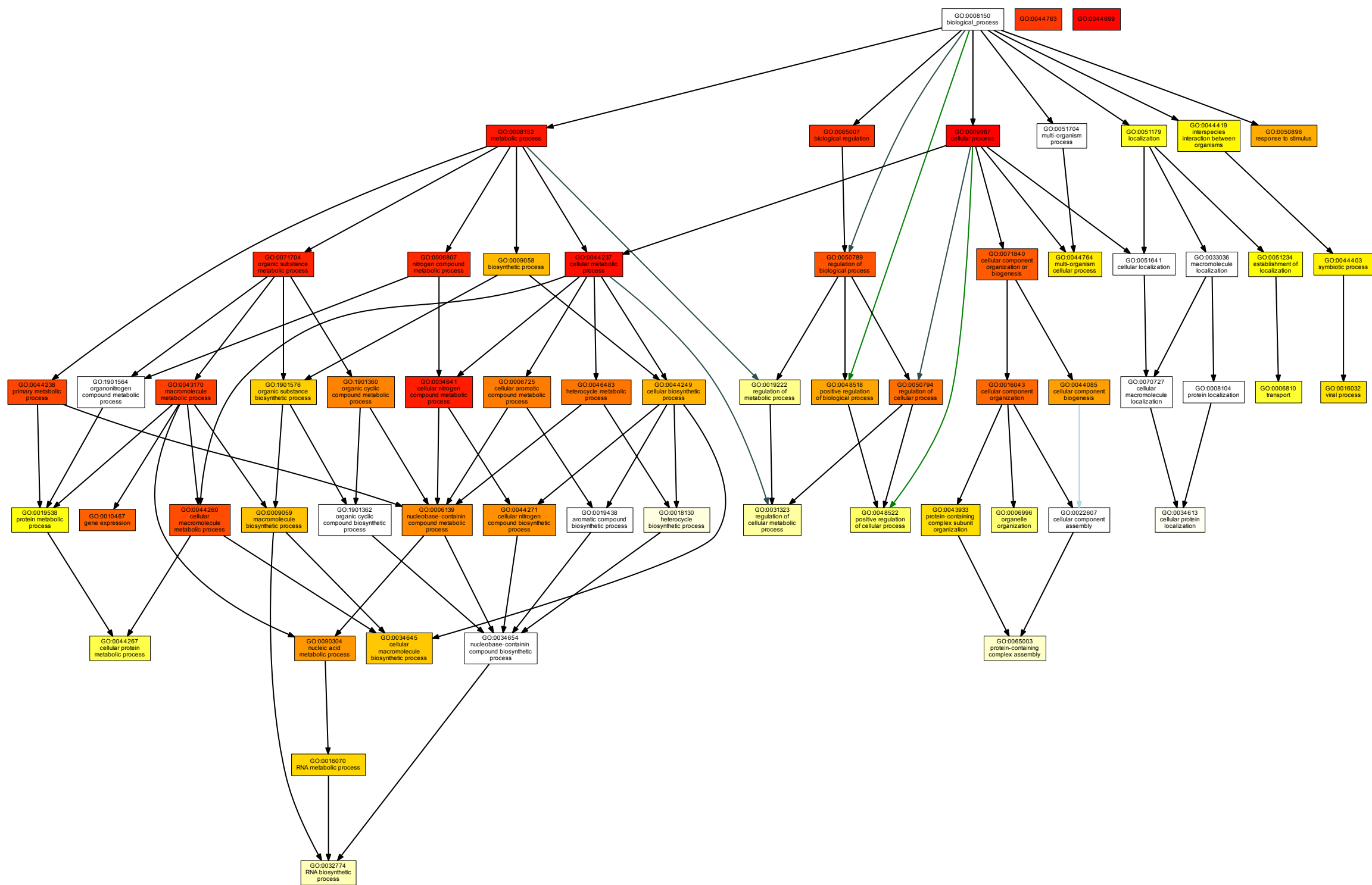

Supplement: Supplemental Material [file KBIE_A_1972780_SM6899.zip › supplementary/Supplementary Figure S4.pdf]

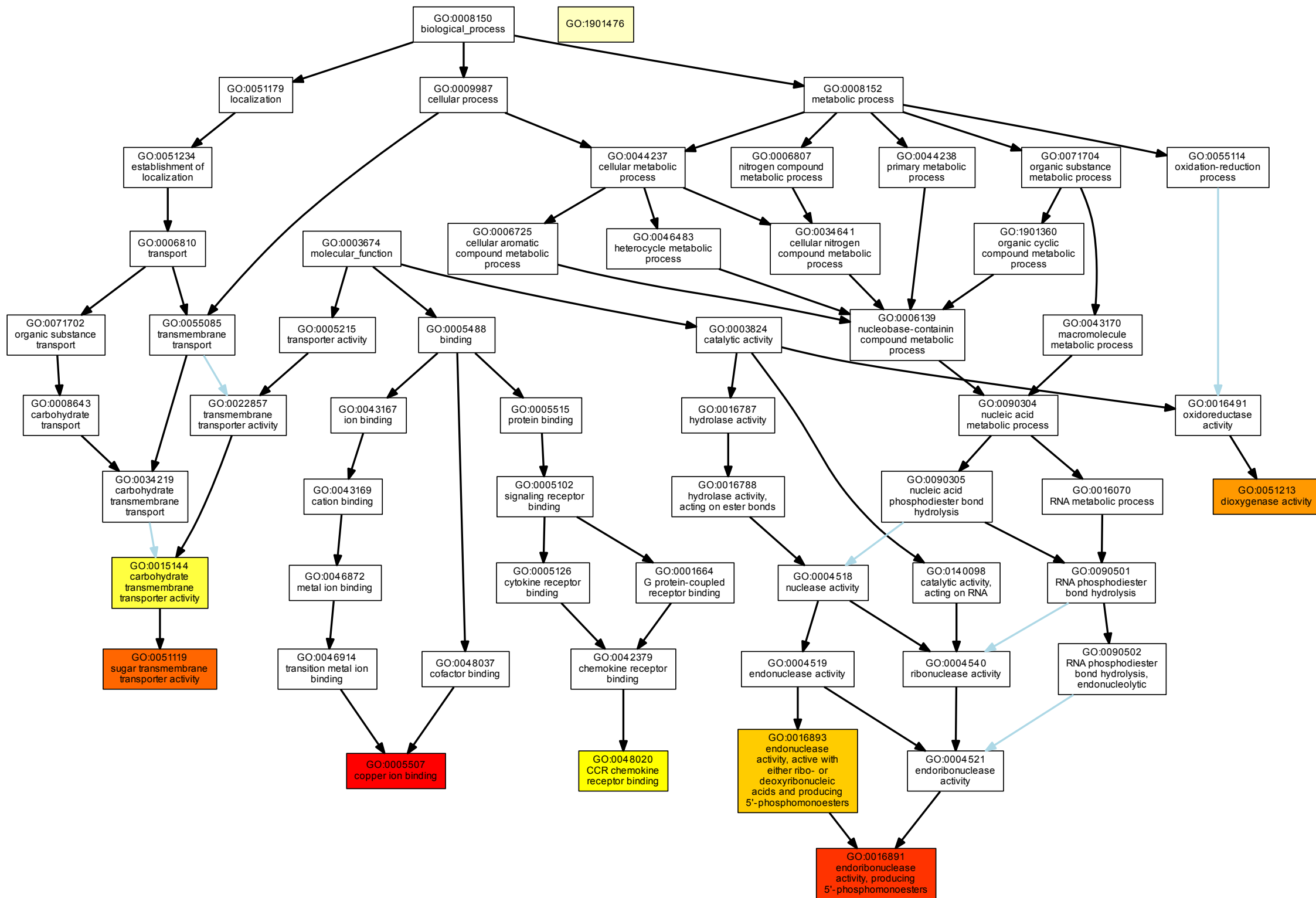

Supplement: Supplemental Material [file KBIE_A_1972780_SM6899.zip › supplementary/Supplementary Figure S5.pdf]

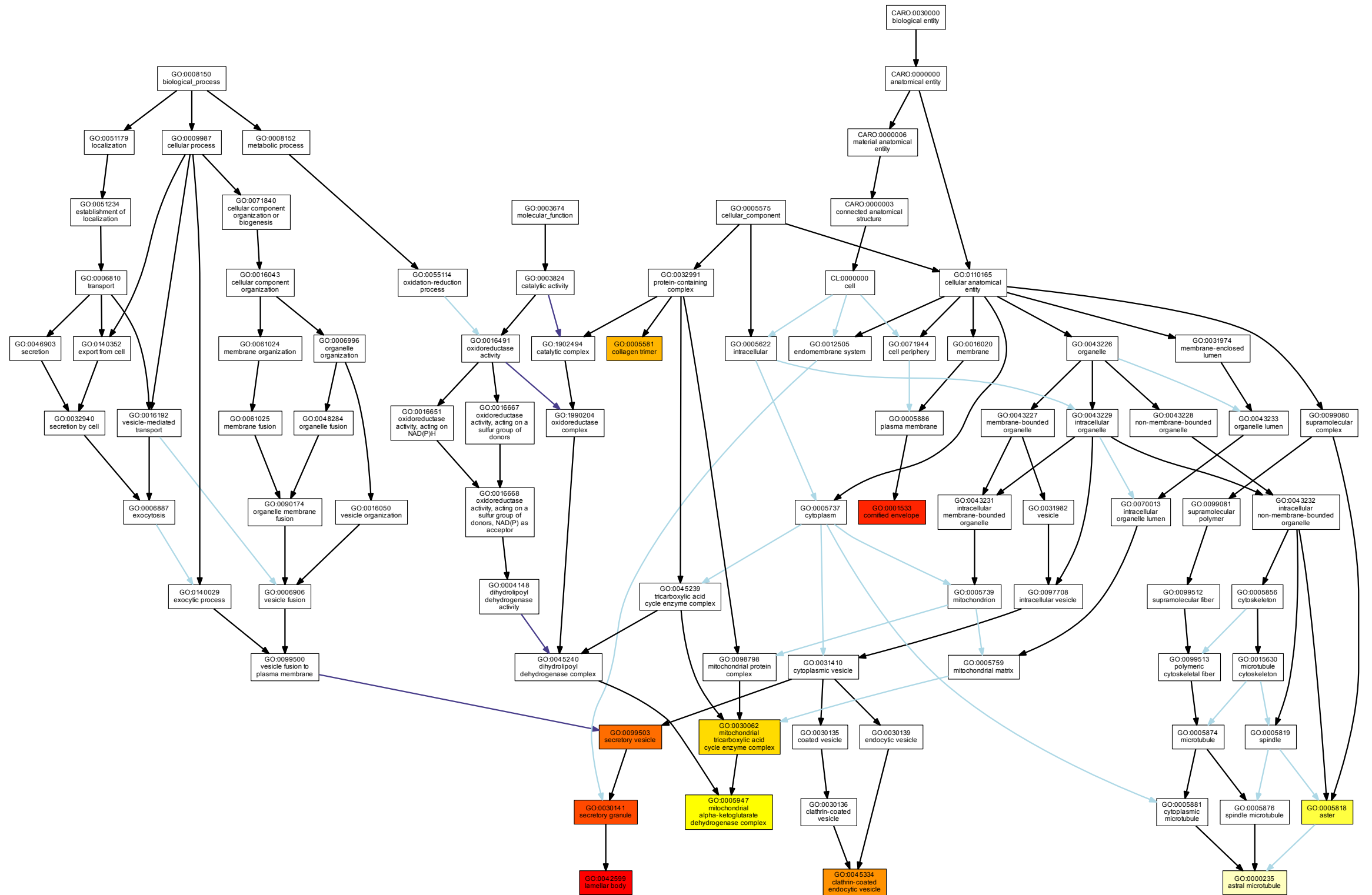

Supplement: Supplemental Material [file KBIE_A_1972780_SM6899.zip › supplementary/Supplementary Figure S6.pdf]

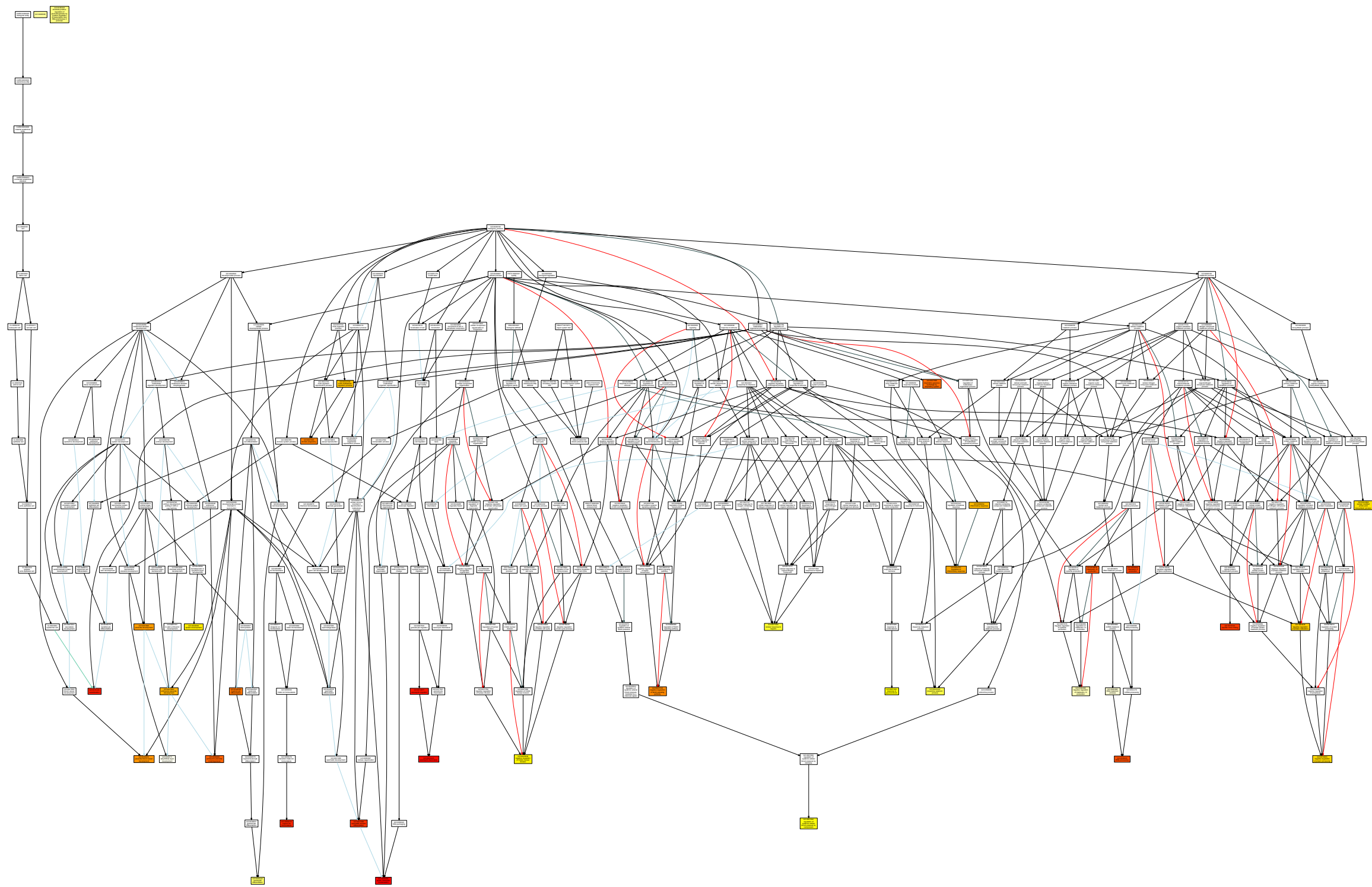

Supplement: Supplemental Material [file KBIE_A_1972780_SM6899.zip › supplementary/Supplementary Figure S7.pdf]
