# Supplementary material for: Exploration of differentially-expressed exosomal mRNAs, lncRNAs and circRNAs from serum samples of gallbladder cancer and xantho-granulomatous cholecystitis patients
Source: Bioengineered. 2021 Sep 4;12(1):6134–43. doi: 10.1080/21655979.2021.1972780 (PMC8806659; doi:10.1080/21655979.2021.1972780)
Supplement: Supplemental Material [file Supplementary Figure S10.pdf › supplementary/Supplementary Figure S10.pdf]

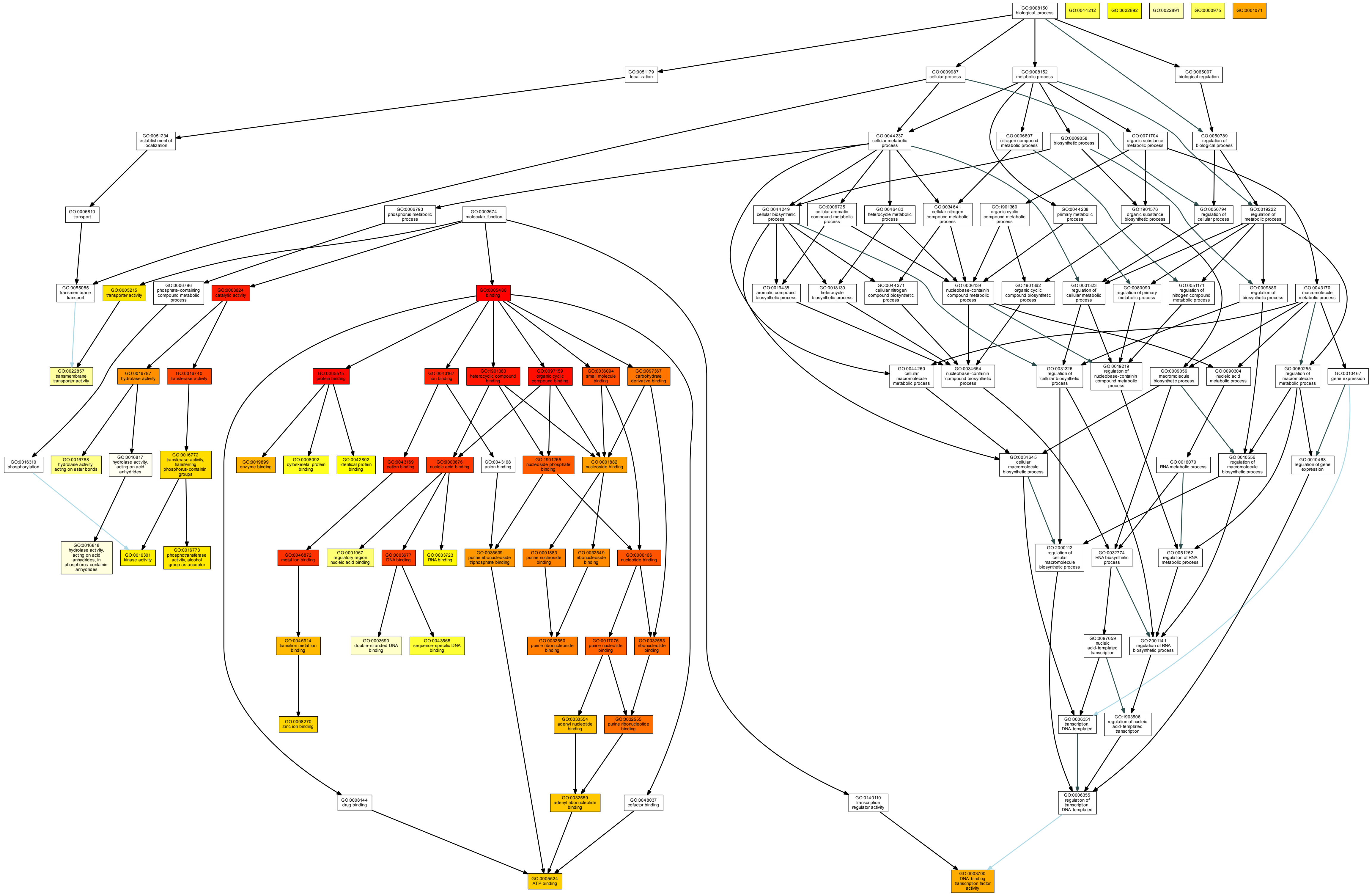

GO:0008150 biological_process
GO:0044212
GO:0022892
GO:0022891
GO:0000975
GO:0001071
GO:0051179 localization
GO:0009987 cellular process
GO:0008152 metabolic process
GO:0065007 biological regulation
GO:0051234 establishment of localization
GO:0044237 cellular metabolic process
GO:0006807 nitrogen compound metabolic process
GO:0009058 biosynthetic process
GO:0071704 organic substance metabolic process
GO:0050789 regulation of biological process
GO:0006810 transport
GO:0006793 phosphorus metabolic process
GO:0003674 molecular_function
GO:0044249 cellular biosynthetic process
GO:0006725 cellular aromatic compound metabolic process
GO:0046483 heterocycle metabolic process
GO:0034641 cellular nitrogen compound metabolic process
GO:1901360 organic cyclic compound metabolic process
GO:0044238 primary metabolic process
GO:1901576 organic substance biosynthetic process
GO:0050794 regulation of cellular process
GO:0019222 regulation of metabolic process
GO:0055085 transmembrane transport
GO:0005215 transporter activity
GO:0006796 phosphate-containing compound metabolic process
GO:0003824 catalytic activity
GO:0005488 binding
GO:0019438 aromatic compound biosynthetic process
GO:0018130 heterocycle biosynthetic process
GO:0044271 cellular nitrogen compound biosynthetic process
GO:0006139 nucleobase-containin compound metabolic process
GO:1901362 organic cyclic compound biosynthetic process
GO:0031323 regulation of cellular metabolic process
GO:0080090 regulation of primary metabolic process
GO:0051171 regulation of nitrogen compound metabolic process
GO:0009889 regulation of biosynthetic process
GO:0043170 macromolecule metabolic process
GO:0022857 transmembrane transporter activity
GO:0016787 hydrolase activity
GO:0016740 transferase activity
GO:0005515 protein binding
GO:0043167 ion binding
GO:1901363 heterocyclic compound binding
GO:0097159 organic cyclic compound binding
GO:0036094 small molecule binding
GO:0097367 carbohydrate derivative binding
GO:0044260 cellular macromolecule metabolic process
GO:0034654 nucleobase-containin compound biosynthetic process
GO:0031326 regulation of cellular biosynthetic process
GO:0019219 regulation of nucleobase-containin compound metabolic process
GO:0009059 macromolecule biosynthetic process
GO:0090304 nucleic acid metabolic process
GO:0060255 regulation of macromolecule metabolic process
GO:0010467 gene expression
GO:0016310 phosphorylation
GO:0016788 hydrolase activity, acting on ester bonds
GO:0016817 hydrolase activity, acting on acid anhydrides
GO:0016772 transferase activity, transferring phosphorus-containin groups
GO:0019899 enzyme binding
GO:0008092 cytoskeletal protein binding
GO:0042802 identical protein binding
GO:0043169 cation binding
GO:0003676 nucleic acid binding
GO:0043168 anion binding
GO:1901265 nucleoside phosphate binding
GO:0001882 nucleoside binding
GO:0034645 cellular macromolecule biosynthetic process
GO:0016070 RNA metabolic process
GO:0010556 regulation of macromolecule biosynthetic process
GO:0010468 regulation of gene expression
GO:0016818 hydrolase activity, acting on acid anhydrides, in phosphorus-containin anhydrides
GO:0016301 kinase activity
GO:0016773 phosphotransferase activity, alcohol group as acceptor
GO:0046872 metal ion binding
GO:0001067 regulatory region nucleic acid binding
GO:0003677 DNA binding
GO:0003723 RNA binding
GO:0035639 purine ribonucleoside triphosphate binding
GO:0001883 purine nucleoside binding
GO:0032549 ribonucleoside binding
GO:0000166 nucleotide binding
GO:2000112 regulation of cellular macromolecule biosynthetic process
GO:0032774 RNA biosynthetic process
GO:0051252 regulation of RNA metabolic process
GO:0046914 transition metal ion binding
GO:0003690 double-stranded DNA binding
GO:0043565 sequence-specific DNA binding
GO:0032550 purine ribonucleoside binding
GO:0017076 purine nucleotide binding
GO:0032553 ribonucleotide binding
GO:0097659 nucleic acid-templated transcription
GO:2001141 regulation of RNA biosynthetic process
GO:0008270 zinc ion binding
GO:0030554 adenyl nucleotide binding
GO:0032555 purine ribonucleotide binding
GO:0006351 transcription, DNA-templated
GO:1903506 regulation of nucleic acid-templated transcription
GO:0008144 drug binding
GO:0032559 adenyl ribonucleotide binding
GO:0048037 cofactor binding
GO:0140110 transcription regulator activity
GO:0006355 regulation of transcription, DNA-templated
GO:0005524 ATP binding
GO:0003700 DNA-binding transcription factor activity
